# The Genetic Puzzle of Familial Atrial Fibrillation

**DOI:** 10.3389/fcvm.2020.00014

**Published:** 2020-02-14

**Authors:** Ahmed A. Y. Ragab, Gustaf D. S. Sitorus, Bianca B. J. J. M. Brundel, Natasja M. S. de Groot

**Affiliations:** ^1^Department of Cardiology, Erasmus University Medical Center, Rotterdam, Netherlands; ^2^Department of Physiology, Institute for Cardiovascular Research, VU Medical Center, Amsterdam, Netherlands

**Keywords:** familial atrial fibrillation, genetics, electrophysiology, arrhythmia, personalized medicine

## Abstract

Atrial fibrillation (AF) is the most common clinical tachyarrhythmia. In Europe, AF is expected to reach a prevalence of 18 million by 2060. This estimate will increase hospitalization for AF to 4 million and 120 million outpatient visits. Besides being an independent risk factor for mortality, AF is also associated with an increased risk of morbidities. Although there are many well-defined risk factors for developing AF, no identifiable risk factors or cardiac pathology is seen in up to 30% of the cases. The heritability of AF has been investigated in depth since the first report of familial atrial fibrillation (FAF) in 1936. Despite the limited value of animal models, the advances in molecular genetics enabled identification of many common and rare variants related to FAF. The importance of AF heritability originates from the high prevalence of lone AF and the lack of clear understanding of the underlying pathophysiology. A better understanding of FAF will facilitate early identification of people at high risk of developing FAF and subsequent development of more effective management options. In this review, we reviewed FAF epidemiological studies, identified common and rare variants, and discussed their clinical implications and contributions to developing new personalized therapeutic strategies.

## Introduction

Atrial fibrillation (AF) is the most common clinical arrhythmia with a rapidly increasing prevalence (1). By 2050, the prevalence of AF is expected to rise to 5.6–15.6 million in the USA (2, 3). AF is associated with an increased risk of complications such as stroke and heart failure (4). Many risk factors are related to the incidence of AF such as age, sex, valvular heart diseases, obesity, alcohol consumption, and hypertension. However, up to 30% of AF cases have no known cardiac pathology or known risk factors (Lone AF) (1). Inherited AF was first reported in the thirties of the last century (5). Recently, the heritability of AF has been recognized and investigated in depth (6–8).

The importance of studying the genetic contribution to AF comes from the high percentage of lone AF cases and the prevalence differences according to gender and among certain ethnic groups. Understanding the heritable component of AF will also facilitate early identification of people at high risk of developing AF later in their lives. For a long time, the limited value of AF animal models especially murine ones obfuscated the investigation of inherited AF. However, after emerging advances in the molecular genetics, many studies identified both rare and common genetic variants related to AF.

In this review, we highlight the findings of familial AF epidemiological studies, the role of both rare and common genetic variants as well as their clinical and therapeutic implications.

## Epidemiological Studies

In the Framingham offspring study, those who had one parent with a history of AF had a 1.8-fold increase in the risk of developing AF. Interestingly, the risk was 3-fold higher in subjects <75 years (9). In the Mayo clinic AF registry, 5% of all patients and 15% of lone AF patients had a family history of AF (10). Among 5,000 Icelanders, the first degree relatives of AF patients were 1.77 times more at risk of developing AF than the general population (7). This relative risk reached 4.67 in patients <60 years. In the Danish twins' study, recurrence risk of AF was 12% for monozygotic twins and 22% for dizygotic twins (8). In another Danish cohort, the incidence rate ratio for lone AF was 3.48 in subjects who had affected first degree relatives and 1.64 in those whose second degree relatives were affected (11).

## Rare Genetic Variants

In the past two decades, many researchers tried to elucidate the genetic base of AF by using different types of studies such as linkage analysis, candidate gene analysis, and whole-genome next-generation sequencing. In 1997, Brugada and his coworkers reported the first genetic locus (10q22-q24) related to AF using the linkage analysis approach (12). A few years later, similar studies reported more genetic loci related to AF, namely 6q14-16, 5p13, 10p11-q21, 20q12-13, and 5p15 (13–16).

In 2003, Chen et al. reported the first gain of function mutation (KCNQ1) in the potassium voltage-gated channel in affected Chinese family. However, the candidate gene analysis was costly, time-consuming and restricted to a small number of scanned genes. Also, the causality effect theory of these variants was not clear as more than 30 different variants have been discovered in potassium channels genes.

## Potassium Channel Variants

Since 2003, many studies reported gain of function mutations in genes coding potassium channels (Table 1). Most of the reported variants were gain of function mutations though, loss of function mutation was also reported. The gain of function mutations result in shortening of the effective refractory period thereby increasing AF vulnerability. Other gain of function mutations have also been identified in KCNE1, KCNE2, KCNE, KCNE5, KCNQ1, and KCNJ2 genes (17, 20, 21, 23, 24, 57–59).

**Table 1:** Summary of gene loci associated with familial atrial fibrillation.

**Gene**	**Locus**	**Mode of inheritance**	**Functional effect**
KCNQ1	11p15.5	Autosomal dominant	Gain of function (17–19)
KCNE1	21q22.1	Autosomal dominant	Gain of function (20)
KCNE2	21q22.1	Autosomal dominant	Gain of function (21)
KCNE3	11q13.4	Autosomal dominant	Gain of function (22)
KCNE5	Xq23	X-linked	Gain of function (23)
KCNJ2	17Q23.1	Autosomal dominant	Gain of function (24)
KCNJ5	11q24.3	Autosomal dominant	Gain of function (25)
KCNJ8	12p12.1	Autosomal dominant	Gain of function (26)
KCNH2	7q36.1	Autosomal dominant	Gain of function (27) Loss of function (28)
KCNA5	12p13.32	Autosomal dominant	Gain of function (29) Loss of function (30)
KCND3	1p13.2	Autosomal dominant	Gain of function (31)
HCN4	15q24.1	Autosomal dominant	Loss of function (32)
MYH6	14q11.2	Autosomal dominant	Loss of function (33)
ABCC9	12p12.1	Autosomal dominant	Loss of function (34)
RYR2	1q43	Autosomal dominant	Gain of function (35)
CACNB2	10p12	Autosomal dominant	Loss of function (36)
CACNA2D4	12p13.33	Autosomal dominant	Loss of function (36)
CAV1	7q31.2	Autosomal dominant	Loss of function (37)
SCN1B	19q13.11	Autosomal dominant	Gain of function (38) Loss of function
SCN2B	11q23.3	Autosomal dominant	Loss of function (39)
SCN3B	11q24.1	Autosomal dominant	Loss of function (40)
SCN4B	11q23.3	Autosomal dominant	Loss of function (41)
SCN5A	3p22.2	Autosomal dominant	Gain of function (42) Loss of function
SCN10A	3p22.2	Autosomal dominant	Gain of function, Loss of function (43, 44)
GATA4	8p23.1	Autosomal dominant	Loss of function (45)
GATA5	20q13.33	Autosomal dominant	Loss of function (46)
GATA6	18q11.2	Autosomal dominant	Loss of function (47)
GJA1	6q22.31	Autosomal dominant	Loss of function (48)
GJA5	1q21.2	Somatic mutation	Loss of function (49)
ZFHX3	16q22.2-q22.3	Autosomal dominant	Loss of function (37)
GREM2	1q43	Autosomal dominant	Gain of function (50)
JPH2	20q13.12	Autosomal dominant	Loss of function (51)
LMNA	1q22	Autosomal dominant	N/A (52)
NUP155	5p13.2	Autosomal dominant	Loss of function (53)
SYNE2	14q23.2	Autosomal dominant	N/A (37)
NKX2-5	5q34	Autosomal dominant	Loss of function (54)
NKX2-6	8p21.2	Autosomal dominant	Loss of function
NPPA	1p36.22	Autosomal dominant	Loss of function (55)
PITX2c	4q25	Autosomal dominant	Loss of function (56)

Mutations of KCNE1 and KCNQ1 affect Iks potassium channels by a gain of function effect which accelerates repolarization and hence, shortens the refractory period. However, mutations of KCNA5 affect Ikur potassium channels but with a loss of function mutation (30, 60). This mutation introduced an alternative mechanism for AF including delayed repolarization and prolongation of the effective refractory period.

## Sodium Channel Variants

In 2005, Olson et al. was the first to report an SCN5A mutation related to AF (61). These reported mutations are encoding α-subunit in Na 1.5 sodium channel. α-subunit gene mutations including genes encoding the four regulatory β-subunits (SCN1B, SCN2B, SCN3B, and SCN4B) are all related to AF (Table 1) (42, 61, 62). Uncovering the underlying mechanisms of these mutations has multiple challenges such as the mixed phenotypes reported, how both loss and gain of function mutations could cause these different phenotypes and the lack of an animal model with pure AF phenotype. Mutations in the SCN10A gene are related to AF. This gene encodes the NA 1.8 sodium channels which is believed to be responsible for late sodium currents and can be modulated by SCN5A level of expression (63).

## Intracellular Calcium Channel Variants

Increased diastolic Ca^2+^ leak is one of the pathophysiological pathways to AF. Phosphorylation of RyR at PKA or CAMKII sites would lead to increased RyR opening probability and increased Ca^2+^ leak from sarcoplasmic reticulum (SR). Recently, a study showed that AF patients have less miRNA-106b-25 cluster with consequent increase in RyR expression and Ca^2+^ leak (64).

## Non-Ion Channel Variants

Gollob et al. described the first three somatic mutations in GJA5 gene related to AF; these mutations are responsible for impaired cell to cell coupling (49). This impairment is caused by depletion of atrial specific connexin 40. Moreover, Christophersen et al. described a germline mutation in the same gene. Mutations in gene encoding atrial natriuretic peptide (ANP) have been reported to be related to AF. It is believed that this mutation in ANP protein would shorten the action potential. In 2008, a mutation in the NUP155 gene encoding nucleoporin of the nuclear envelope was discovered. This mutation leads to alteration in nuclear envelope permeability. Many mutations in transcription factors genes have been reported to be related to AF such as NKX2-5, PITX2, ZFHX3, GATA4, GATA5, and GATA6 genes. GATA and PITX2 genes affect the development of the pulmonary venous myocardium which is involved in the initiation of AF (Table 1). Several studies reported an increased risk of AF with polymorphism of RAAS system genes encoding angiotensin converting enzyme inhibitor, angiotensin gene promotor, and angiotensinogen (24, 65).

## Limitations Of *in vitro* Methods

*in vitro* methodologies for functionally characterizing the role of ion channels variants have drawbacks. For instance, AF cell lines continuously proliferate and are affected by rapid maturation, increased number of cells, and disorganized three-dimensional structure. In addition, not all areas within cell lines have the same metabolic activity. The evolving induced pluripotential stem cells is one step closer to the optimal *in vivo* conditions such as conduction properties, contraction and relaxation velocity, action potential duration, and repolarization fraction. Repolarization fraction is a parameter to distinguish between atrial and ventricular like human induced pluripotent stem cells (hiPSCs) and it is calculated based on the following equation: (APD90–APD50)/APD90), APD90; is action potential duration at 90% repolarization and APD 50 is action potential duration at 50% repolarization. However, these type of cells are electrophysiologically different from adult atrial cardiomyocytes in respect to Ca^2+^ handling and the predominance of ventricular like cells; ventricular contribution to the cell population can be minimized to <10% by using timed retinoic acid exposure.

## Murine Models

In recent decades, murine models have drawn the attention of many investigators attempting to decode electrophysiological mechanism underlying AF. Murine models were considered a good candidate because of the conservation of development and signaling pathways between homo sapiens and mice, the ease of genetic manipulation, and rapid maturation.

Potassium channels mutation models have been studied such as the knockout models for KCNE1and SK2 channels (66–69). Moreover, sodium channel genes have been a target for transgenic models. ΔKPQ-SCN5A models showed more susceptibility to atrial arrhythmia (70–74). SCN3B subunit knockout models also showed conduction disturbances (75). Non-ion channels models also showed promising results such as connexin 40 and 43 models (76–78), Ankyrin B (79), and PITX2 (80). Knock out mice of spinophilin-1 leads to increased RyR phosphorylation and increases Ca^2+^ leak (81). The same results were also shown in junctophilin and FKBP-12.6 knock out models (51, 82).

Despite the value of these murine models, they have several limitations. One of the main limitations of these models is that AF was always induced in a non-physiological way. Other factors involved in clinical AF such as environmental factors, diet, and abuse of toxic substances were omitted. Although there is similarity in signaling pathways between mice and humans, there are important differences in heart rate, ion currents, calcium handling, and predominant myosin isoform.

## Genome Wise Association Studies (GWAS)

In 2007, the first GWAS study on AF was published. By using a *p*-value of < 5 × 10^−8^ to minimize false positives, variant frequencies were compared between affected and non-affected subjects. The first detected locus was on chromosome 4q25 (83). However, this locus is in a non-coding area; studies revealed its role in regulating the closest gene (PITX2). This gene is essential for cardiac development and suppression of a sinus node development in pulmonary vein myocardium (left-right asymmetry). PITX2 knockout mice model showed a decrease in sodium and potassium channels expression and caused a conduction block at the atrioventricular node (84). Herraiz-Martínez et al. recently investigated whether chr4q25 risk variants alter the intracellular calcium homoeostasis. Patients carrying the rs13143308T risk variant show increased SERCA2a expression, SR calcium load, and RyR2 phosphorylation. These changes lead to excessive calcium release and a higher risk for AF (85).

In 2009, a novel locus on chromosome 16q22 was described in a cohort of European descent (86). The closest single nucleotide polymorphism (SNP) to this locus was intron to the zinc finger homeobox 3 (ZFHX3). This motif binding factor is required for regulation of the Pituitary-specific positive transcription factor 1 (POU1F1) which interacts with the PITX2 gene. In 2010, Ellinor et al. described a novel locus on chromosome 1q21. This SNP is located near KCNN3 gene which encodes voltage-independent calcium-activated potassium channel protein. These SK3 channels are essential for the repolarization phase of the cardiac action potential (87). These SK3 channels are also located in the inner mitochondrial membrane and opening of these channels using agonists have a protective effect against oxidative stress-induced injury resulting from Ca^2+^ overload (88).

Chromosome 15q24 also contained a locus related to AF and sinus node dysfunction. The closest gene to this SNP was HCN4 which encodes channels proteins regulating funny current of the sinoatrial node in the left atrium (89). Recently, two SNPs were discovered in the Japanese population on chromosome 12q24. The first SNP is located near NEURL gene. Knocking out this gene in zebrafish lead to action potential prolongation. The other SNP was intronic to CUX2 gene, however, the mechanism leading to AF is not clear yet.

An AF GWAS risk SNP on chr14q23 in the SYNE2 encodes nesprin-2 which is part of nuclear outer membrane and sarcomere (90, 91). Another non ion channel gene showed AF related SNP on chromosome 7q31, this locus is intronic to CAVI (caveolin-1) which has a role in repolarization phase of action potential and also has a structural role by regulating TGF-β-1 and fibrosis (92).

Recently, many studies investigated the role of cytoskeletal proteins in the pathogenesis of FAF. Two Islandic cohorts reported two novel SNPs in MYH6 and MYL4 genes (93, 94). MYH6 encodes the alpha myosin heavy chain subunit. Mutations in this subunit have been reported to affect cardiac contractility and muscles fibers integrity (95, 96). MYL4 encodes the essential myosin light chain subunit which is known as atrial light chain1. *In vitro* experiments on zebrafish with mutant MYL4 revealed loss of cardiac contractility and absence of sarcomere structure (97, 98). Another study supported the role of myocardial structure in FAF by the discovery of a missense variant in the PLEC gene (99). This gene encodes a cross-linking protein (plectin) which has a role in keeping the integrity of cardiac muscles. These studies suggest a strong role of cytoskeletal proteins in the pathogenesis of AF. A recent large GWAS meta-analysis showed that AF is associated with variants in 18 structural genes and also variants in 13 genes with a cardiac fetal developmental role such as ARNT2 and EPHA3 (100). This could explain the pathophysiology of AF as a result of atrial cardiomyopathy via cardiac structural remodeling either during fetal development or during adult life.

Another large GWAS study identified 134 AF associated loci among 93,000 AF cases and more than 1 million referents (101). This study showed that TBX3, TBX5, and NKX2-5 genes encode transcriptional factors that regulate development of the cardiac conduction system. This study also highlights the overlap between AF and other atrial arrhythmias and the pleiotropy of genes which are responsible for cardiac morphology and function. Nielsen et al. showed the relationship between AF and cardiac development and suggested that AF variants play a role in the developing heart or in reactivating fetal genes or pathways during adulthood as a response to stress and remodeling (100).

Despite the revolutionary output of GWAS studies, this approach of investigating heritability of FAF has several limitations. A large number of detected loci has only explained a small fraction of the missing heritability. This fact limits the clinical usage of outcomes of GWAS studies and urges the need for studies investigating gene-gene and gene-environment interactions. Another challenge is that approximately 80% of the discovered SNPs are in non-coding regions of the genome and this requires additional research to explore the exact causal variant by deploying techniques such as fine mapping, functional analyses, and evolutionary genetics.

## Clinical Implications

There is no doubt that FAF is part of the uprising field of personalized medicine. Technological advances in genetics and a large amount of newly available data have encouraged many researchers to investigate the possible clinical value of this data to develop more efficient prediction models and personalized management strategies. The ORBIT-AF registry showed that FAF patients experienced more symptoms than non-FAF patients. However, there was no difference between the two groups regarding AF recurrences, hospitalization rate, complications, and all-cause mortality (102, 103). On the other hand, risk stratification based on genotype showed promising results. Husser et al. and Shoemaker et al. showed that patients with 4q25 SNP rs2200733 had an increased risk of developing recurrent AF after ablation (104, 105). Another study showed that AF patients with the same 4q25 SNP also had higher risks of developing AF recurrences after direct current cardioversion (HR:2.1, 95% CI: 1.21–3.3; *P* = 0.008) (106). The main limitations of these results are the small sample sizes and using the time to the first symptomatic episode which is a poor quantitative metrics for AF. Time to the first symptomatic AF episode does not take into account the frequency and length of AF episodes. Advances in continuous rhythm monitoring devices and AF detection algorithms will facilitate using AF burden as a more realistic, accurate and quantitative parameter for AF and also as a surrogate outcome after treatment. The effect of genotype on the success of ablative therapy was tested; likewise, response to antiarrhythmic drugs. Parvez et al. showed that the SNP rs10033464 at 4q25 is an independent predictor for success in rhythm control in both discovery and validation cohorts. Furthermore, they showed this same SNP is a predictor for AF recurrence in the same cohorts (107). Another study showed that flecainide potency is increased in AF patients with β1AR Arg389Arg genotype (108). Also, AF patients with the same genotype have a better response to rate control therapy and required lower doses of these drugs (109). One of the main limitations of these studies is the lack of randomization. Data were analyzed retrospectively and drug response was evaluated a priori without knowledge of the genotype.

Few studies tried to implement genotype into prediction models of *de novo* AF. In 2013, AF-genetic risk score (AF-GRS) was introduced. This score consisted of 12 risk alleles in nine loci. They investigated the predictive value of this score in 20000 females without cardiovascular disease at baseline. Adding this score to the main prediction model increased the area under the curve to (0.74) (110). In 2014, Tada and his colleagues showed that multiple single nucleotide polymorphisms can improve the prediction to develop AF and ischemic stroke. GRS score showed a potential value as an indicator for anticoagulant therapy (111). The main limitation of these studies is their lack of external validity to other ethnic groups such as Africans or Asians.

For postoperative AF, few studies have tried to replicate this approach but results are still controversial to improve prediction models performance as these studies lacked large sample sizes and did not use continuous ECG monitoring to identify AF episodes (112–114). In 2016, Lin and his colleagues investigated if gene-gene interaction would affect AF susceptibility. However, this study could not find any significant association and a larger cohort containing participants from other ethnic groups is indeed justified (115).

## Translational Challenges

Translating the advances achieved in genetic technology into clinical practice still has many limitations with respect to genetic based prediction models and personalized therapeutic strategies.

Firstly, prediction models still have insufficient discriminative ability between low and high-risk individuals for several reasons such as testing small number of variants, potential gene-gene interactions, and gene-environment interactions. Moreover, the cost and logistic aspects have to be considered while moving this prediction model into clinical use. Secondly, applications of pharmacogenetics guided therapy are limited.

Another limitation is that pathophysiological pathways underlying AF genetic variants are not clear which delays attempts to target certain pathways caused by specific genetic variants. The multifactorial complex nature of AF could also limit the efficacy of any new drug development. In addition, involvement of multiple genetic variants in a patient is more challenging for a personalized efficient treatment strategy.

## Future Directions

Despite the advances in our understanding of FAF, there are still many challenges and questions to be addressed. Firstly, large cohorts are needed to study the effect of gene-gene and gene-environment interactions on AF. These cohorts should consider larger sample sizes, participation of non-European ancestry and analyzing interactions between more than two variants. Secondly, randomized controlled trials are needed to validate the effect of genotype guided treatment strategies. Advances in rhythm monitoring devices and rhythm detection algorithms are needed in addition to using AF burden as a reliable parameter to quantify AF.

Larger cohorts are needed to investigate the effect of genotype guided prediction models of AF incidence, AF complications and mortality. Last but not least, large and effective screening studies for families with FAF is advised to uncover part of the missing heritability of FAF. For instance exome sequencing and whole genome sequencing projects would discover more missing rare and structural variants which GWAS studies cannot identify.

## Conclusion

Genetic basis and heritability of AF is part of the complexity of this arrhythmia and a lot of progress has been achieved in many aspects such as risk stratification for AF, identification of novel therapeutic targets, and genome-based prediction models. There is no doubt that better understanding of AF heritability will not only improve AF prediction models but will also be the next step toward more efficient personalized treatment strategies.

## Author Contributions

AR and GS: drafting article and interpretation. BB and NG: critical revision and approval of article.

### Conflict of Interest

The authors declare that the research was conducted in the absence of any commercial or financial relationships that could be construed as a potential conflict of interest.
